# Current Animal Models of Alzheimer’s Disease: Challenges in Translational Research

**DOI:** 10.3389/fneur.2014.00182

**Published:** 2014-09-29

**Authors:** Mar Cuadrado-Tejedor, Ana García-Osta

**Affiliations:** ^1^Neurobiology of Alzheimer’s Disease, Neurosciences Division, Center for Applied Medical Research, CIMA, University of Navarra, Pamplona, Spain; ^2^Department of Anatomy, University of Navarra, Pamplona, Spain

**Keywords:** Alzheimer’s disease, AD-mouse models, neuronal loss, multifactorial origin, therapeutics

In their article, Franco and Cedazo-Minguez open the debate on why it is difficult to translate successful preclinical research in Alzheimer’s disease (AD) mouse models into clinical practice (1). Here, we discuss some aspects that should be taken into account regarding the main discrepancies that exist between the current animal models and the disease in humans.

The translation of findings from bench to clinically relevant therapies is very complex. In fact, despite a full preclinical and clinical trial package, the large majority of drugs with initial phases based on translational-laboratory-based discoveries actually fail to complete the development process. A lack of efficacy, side-effects, inappropriate doses, and pharmacokinetics are just a few of the various reasons for this failure. Furthermore, the preclinical disease models on which new drugs are tested may not always be predictive of the effect of the agent in the human disease state (2). Could this be, as Franco and Cedazo-Minguez suggest, one of the major concerns in translational research in the case of AD?

On the one hand, one of the main points to consider is probably the fact that most of the AD-mouse models do not present the extensive neuronal loss observed in the brain of AD patients. At the moment of clinical diagnosis, most of the patients with AD-type dementia already have a Braak stage V or VI with a substantial synaptic and neuronal loss (3). Nevertheless, the loss of synapses is the best correlate of the cognitive impairment in patients with AD (4, 5). The synapse loss, which predates neuronal death in the human condition, is present in most of these mouse models, suggesting that they may represent the prodromal phase of the disease. Several authors have proposed that in the human condition, as a compensatory response, an enlargement of remaining synapses may occur, allowing the system to respond properly (6, 7). This could be one of the reasons why progression from early-phase to symptomatic stages in AD takes such a long time. It has been suggested that this “silent” period of the disease can even last for decades (8). Therefore, many of the therapies assayed on the AD models that are ineffective in people with the already established pathology might possibly be effective in preventing or delaying disease progression toward dementia. Although none of the animal models may represent the best option for evaluating novel therapeutic approaches for mild to moderate AD cases, they might be the first step in evaluating drugs that could reverse the synapse loss that underlies the “silent” phase of the disease. In animal models, the synapse loss underlies the memory deficits observed with the behavior tasks used for testing memory function. Therefore, therapeutic approaches for reversing memory deficits in AD-mouse models through the enhancement of the synaptic function and/or spine density might be of great value for treating the memory decline that also occurs in patients with “mild cognitive impairment” (MCI), a term proposed by Petersen et al. as a new diagnostic entity for the transition between normal aging and AD dementia (9). Ultimately, since the AD drug development mainly motivated by the amyloid hypothesis had frightening results, the latest idea is that other pathways, which are not directly linked to Aβ, should be explored. In this context, phosphodiesterase-inhibitors, already on the market for other clinical uses (10) or epigenetic drugs (11) as potential memory enhancers could be a reliable option. Moreover, it is also important to note that all the AD therapies assayed in different clinical trials that could not continue on to subsequent phases due to the appearance of side-effects or those that have failed because the dose assayed in human trials had not been properly established, should also be carefully reviewed. Investing in the improvement of current drugs that have already been assayed and/or in drug-repurposing might be of special use in the case of AD.

On the other hand, it should be taken into account that sporadic forms of AD have a multifactorial origin, with many different risk factors contributing to AD progression. Reducing any one of them by acting on/or improving the neural environment of the brain of these AD-mouse models (by antioxidants, vitamins, cognitive enhancers, vasodilators, etc.) may be sufficient for ameliorating the incipient AD-phenotype of the models. Moreover, different mice strains should be used for modeling human-like environment factors because the use of inbred strains with a common genetic background, housed in a controlled environment, eliminates most of the variability that exists in the human condition. Some researchers have proposed that benefits with a new therapeutic intervention should be demonstrated in at least two different animal models and replicated by independent laboratories before beginning human experimentation (2). In addition, it is important to highlight that among the risk factors, aging, which is the most important one, is not always present in the preclinical studies carried out on animal models. The overexpression of familial AD-genes in these models accelerates the onset of the AD-phenotype, with amyloid plaques and synaptic deficits appearing even when the animals are 2–4 months old. The possible advantage of using these early models, with their early onset of symptoms, has the disadvantage of compromising the age factor. Therefore, the use of late-plaque models (i.e., Tg2576, PDAPP, TgAPP23) for preclinical studies could be more accurate than using early plaque models (12). Figure 1 shows the main AD features developed in Tg2576 mice (a late-plaque model) over time; this model has been used in different studies in our laboratory over the last 10 years.

**Figure 1:**
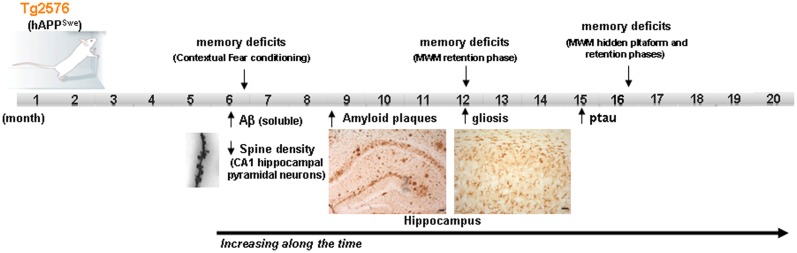
**Development of the different AD signs in Tg2576 mice over time**. Scheme showing the time point of main AD features apparition in Tg2576 mice. At the age of 16 month, although no neuronal loss is presented in the brain of Tg2576 mice, the AD-phenotype is well established. MWM Morris water maze.

In summary, although we agree with most of the statements made in the review by Franco and Cedazo-Minguez, since animal models are indeed mandatory for preclinical studies, we consider that in the case of AD, the model selected (a late onset model with an established phenotype) and the appropriate dosage regimen may be critical for the successful translation of experimental drugs to humans.

## Conflict of Interest Statement

The authors declare that the research was conducted in the absence of any commercial or financial relationships that could be construed as a potential conflict of interest.
